# The Role of the p38 MAPK Signaling Pathway in High Glucose-Induced Epithelial-Mesenchymal Transition of Cultured Human Renal Tubular Epithelial Cells

**DOI:** 10.1371/journal.pone.0022806

**Published:** 2011-07-29

**Authors:** Zhi-Mei Lv, Qun Wang, Qiang Wan, Jian-Gong Lin, Meng-Si Hu, You-Xia Liu, Rong Wang

**Affiliations:** Department of Nephrology, Provincial Hospital Affliated to Shandong University, JiNan, China; Institut National de la Santé et de la Recherche Médicale, France

## Abstract

**Background:**

Epithelial-mesenchymal transition of tubular epithelial cells, which is characterized by a loss of epithelial cell characteristics and a gain of ECM-producing myofibroblast characteristics, is an essential mechanism that is involved in tubulointerstitial fibrosis, an important component of the renal injury that is associated with diabetic nephropathy. Under diabetic conditions, p38 MAPK activation has been reported in glomeruli and mesangial cells; however, studies on p38 MAPK in TECs are lacking. In this study, the role of p38 MAPK in AP-1 activation and in the EMT in the human proximal tubular epithelial cell line (HK-2) under high glucose concentration conditions is investigated.

**Methodology/Principal Findings:**

A vector for small interfering RNA that targets p38 MAPK was constructed; the cells were then either transfected with p38 siRNA or pretreated with a chemical inhibitor of AP-1 and incubated with low glucose plus TGF-β1 or high glucose for 48 h. Cells that were not transfected or pretreated and were exposed to low glucose with or without TGF-β1 or high glucose for 48 h were considered to be the controls. We found that high glucose induced an increase in TGF-β1. And high glucose-induced p38 MAPK activation was inhibited by p38 siRNA (P<0.05). A significant decline in E-cadherin and CK expression and a notable increase in vimentin and α-SMA were detected when exposed to low glucose with TGF-β1 or high glucose, and a significant raise of secreted fibronectin were detected when exposed to high glucose; whereas these changes were reversed when the cells were treated with p38 siRNA or AP-1 inhibitor (P<0.05). AP-1 activity levels and Snail expression were up-regulated under high glucose conditions but were markedly down-regulated through knockdown of p38 MAPK with p38 siRNA or pretreatment with AP-1 inhibitor (P<0.05).

**Conclusion:**

This study suggests that p38 MAPK may play an important role in the high glucose-induced EMT by activating AP-1 in tubular epithelial cells.

## Introduction

Glomerular mesangial expansion and podocyte loss are important early features of diabetic nephropathy, and tubulointerstitial injury and fibrosis are critical for the progression of diabetic nephropathy to kidney failure. It has been shown that tubulointerstitial fibrosis (TIF) is a more consistent predictor of functional impairment than glomerular damage. Accumulating evidence suggests that TECs play a pivotal role in TIF by undergoing EMT, which increases extracellular matrix (ECM) synthesis [1]–[3]. The EMT of tubular epithelial cells is proposed as an orchestrated, highly-regulated process that consists of four key steps [4]: (1) loss of epithelial cell adhesion; (2) de novo α-SMA expression and actin reorganization; (3) disruption of the tubular basement membrane; and (4) enhanced cell migration and invasion.

The transforming growth factor-β (TGF-β) family of secreted factors regulates various biological processes, including cell proliferation, differentiation and apoptosis [5]. TGF-β can induce mesenchymal transdifferentiation in epithelial and endothelial cells through various signal pathways[6]. Thus in our study, to further confirm whether high glucose could induce EMT in TECs, we observed expression of TGF-β1 under high glucose conditions, given the fact that TGF-β is the key inducer of EMT [7], [8].

p38 MAPK is a member of the MAPK family and is essential for the regulation of many cellular processes, including inflammation, cell differentiation, cell growth and cell death [9]–[11]. p38 MAPK mediates the signals that are relevant to thedevelopment of diabetic nephropathy. It is thought that p38 MAPK is a signal transducer in the underlying diabetic nephropathy pathways, and it has been proposed that the agents that inhibit the p38 MAPK signaling pathway may reduce the formation of the ECM in the glomerular mesangium and block the thickening of the glomerular basement membrane, thus preventing the development of diabetic nephropathy [12]. There is a wealth of data that supports the central role of the p38 MAPK signaling pathway in high glucose-induced cell damage [13]–[17]. Recent in vitro studies have shown that high levels of glucose can activate the p38 MAPK signaling pathway in renal cells and induce the phosphorylation of p38 MAPK, which promotes the production of fibronectin by the mesangial cells [18]–[21]. In addition, there is enough evidence that TGF-β signals through MAPKs [22], and the activation of p38 MAPK is required in TGF-β-induced EMT in mammary epithelial cells [23]. Therefore, we decided to examine whether the p38 MAPK signaling pathway contributes to the EMT that is induced by high glucose in human proximal tubular epithelial cells.

AP-1, which is a transcription factor, is a heterodimeric protein that is composed of proteins of the c-Fos, c-Jun, ATF and JDP families. AP-1 regulates gene expression in response to a variety of stimuli, including cytokines, growth factors, stress, and bacterial and viral infections [24]–[28]. It has been shown that a tubular overactivation of AP-1 and a simultaneous up-regulation of certain proinflammatory and profibrogenic genes are markers of progressive renal disease in humans [29]. The activation of AP-1 through dimerization that is mediated by leucine zippers is known to be promoted by a variety of upstream protein kinases [30]. Upstream signaling pathways, which include mitogen-activated protein kinases (MAPKs), regulate the transcriptional activity and the half-life of certain proteins and produce AP-1 dimers of different transcriptional specificity [31]. Because MAPKs have been shown to be involved in a crucial step in the up-regulation of AP-1 activation in various types of cultured cells via multiple mechanisms, we attempted to examine the possible involvement of p38 MAPK, the major upstream kinase, in AP-1 activation in HK2 cells.

The purpose of this study was to determine the role of p38 MAPK in high glucose-induced AP-1 activation and in the EMT in the HK-2 cells. A vector for small interfering RNA that targets p38 MAPK was constructed; the cells were then either transfected with p38 siRNA or pretreated with a chemical inhibitor of AP-1 and incubated with low glucose (5.5 mM glucose) plus TGF-β1 or high glucose (30 mM glucose) for 48 h. Cells that were not transfected or pretreated and were exposed to low glucose (5.5 mM glucose) with or without TGF-β1 or high glucose (30 mM glucose) for 48 h were considered to be the controls. In this study, it was found that the activation of p38 MAPK under high glucose conditions led to EMT in HK-2 cells. In addition, p38 siRNA partially suppressed α-SMA and vimentin expression and inhibited fibronectin synthesis. The activation of AP-1 in the high glucose-induced EMT and its partial abrogation by p38 siRNA were also shown. Furthermore, p38/AP-1 promoted the EMT via suppression of E-cadherin through up-regulated Snail expression.

## Results

### SiRNA silenced p38 MAPK in HK-2 cells cultured with high glucose

**Figure 1:**
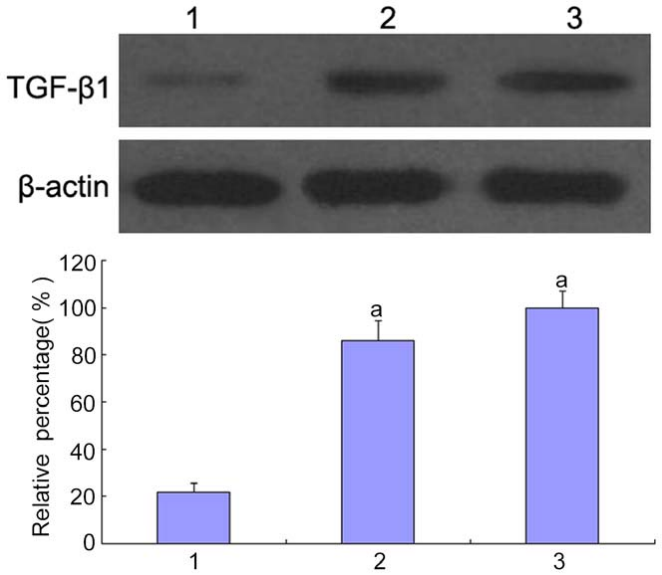
The expression of TGF-β1 in HK-2 cells by Western blot. TGF-β1 was measured by Western blot of the HK-2 cells cultured in 5.5 mM glucose (lane 1), 30 mM glucose for 24 h (lane 2), 30 mM glucose for 48 h (lane 3). ^a^P<0.05 vs. 5.5 mM glucose.

**Figure 2:**
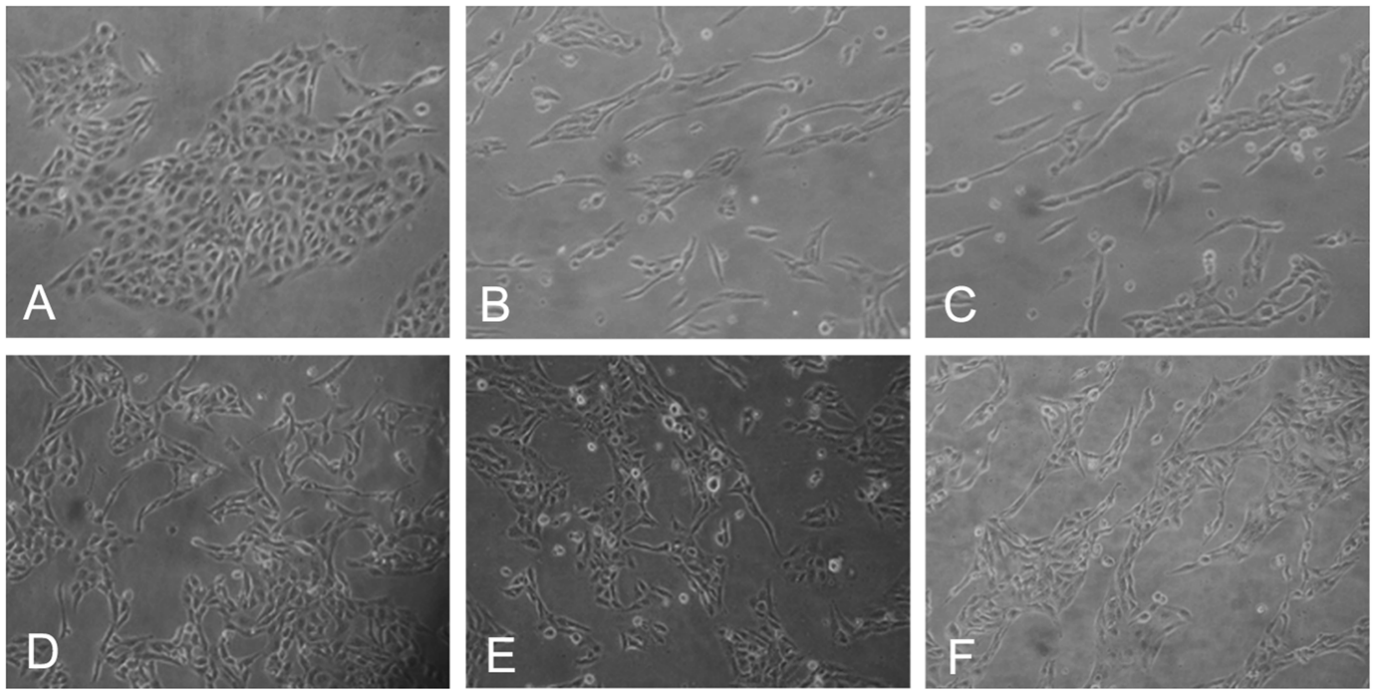
Inverted microscope analysis of the HK-2 cells cultured under different conditions. (A) A typical epithelial cuboidal shape of the HK-2 cells cultured in 5.5 mM glucose condition were shown, with the characteristic cobblestone morphology. (B–C)Morphological changes of the HK-2 cells. The cells became more elongated, less adhered and lost their apical-to-basal polarity after treated with 30 mM glucose(B) or 30 mM glucose+Cont siRNA(C).(D–F)Changes of the cells were reversed, which were exposed to 30 mM glucose+p38 siRNA for 24 h(D) or for 48 h(E) or 30 mM glucose+AP-1 inhibitor(F).

**Figure 3:**
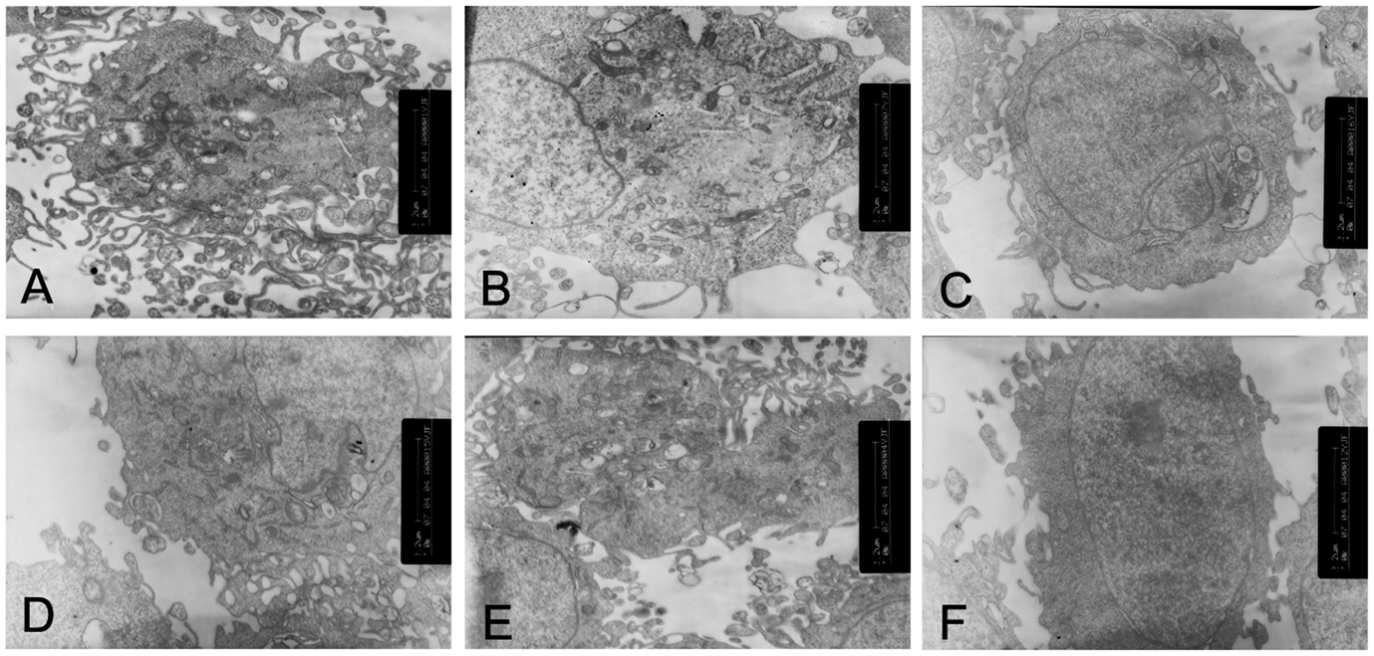
Transmission electron microscopic analysis of the HK-2 cells cultured under different conditions. (A–C)The HK-2 cells under 30 mM glucose (B) or 30 mM glucose+Cont siRNA (C) showed decreased number of microvilli, mitochondria and increased volume density of rough endoplasmic reticulum compared to that of the cells cultured in 5.5 mM glucose(A). (D–F)Changes of the cells were reversed,which were cultured in the 30 mM glucose+p38 siRNA for 24 h(D) or 48 h(E) or 30 mM glucose+AP-1 inhibitor(F).

**Figure 4:**
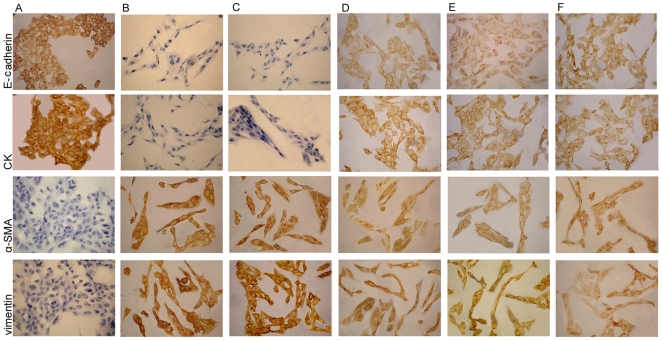
Expression of E-cadherin, CK, α-SMA, vimentin in HK-2 cells by immunochemistry. (A–C)The HK-2 cells under 30 mM glucose(B) or 30 mM glucose+cont siRNA(C) showed a loss of CK and E-cadherin and an increase of α-SMA and vimentin expression compared to that of the cells under 5.5 mM glucose condition(A).(D–F)These changes were prevented by exposed to 30 mM glucose+p38siRNA for 24 h(D) or 48 h(E) or+AP-1 inhibitor(F).

**Figure 5:**
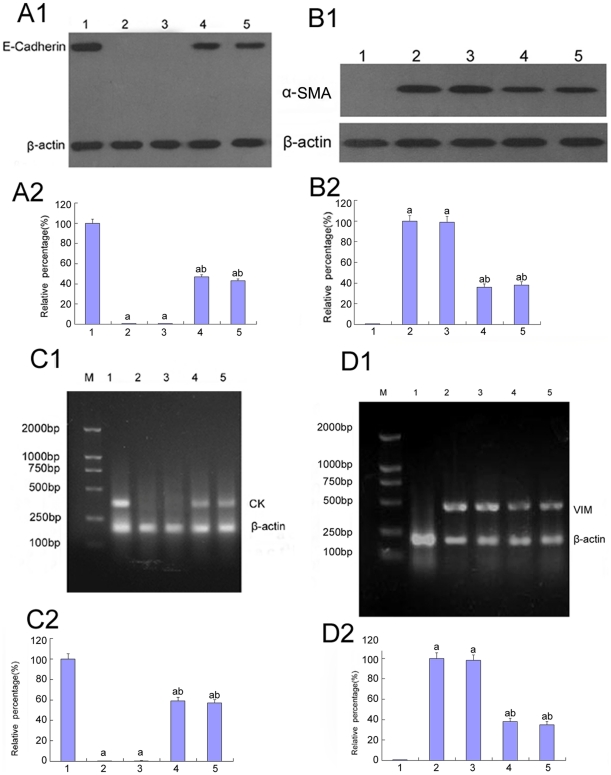
Expression of E-cadherin, CK, α-SMA, vimentin in HK-2 cells by Western blot and RT-PCR analysis. (A–B)E-cadherin expression(A1,A2) and α-SMA(B1,B2) were analyzed by Western blot of the HK-2 cells cultured in 5.5 mM glucose (lane 1), 30 mM glucose (lane 2), 30 mM glucose+Cont siRNA(lane 3), 30 mM glucose+p38 siRNA for 24 h(lane 4), 30 mM glucose+p38 siRNA for 48 h(lane 5).(C–D)mRNA of CK (C1,C2) and vimentin(D1,D2) were analyzed by RT-PCR of the HK-2 cells cultured in 5.5 mM glucose (lane 1), 30 mM glucose (lane 2), 30 mM glucose+Cont siRNA(lane 3), 30 mM glucose+p38 siRNA for 24 h(lane 4), 30 mM glucose+p38 siRNA for 48 h(lane 5).Values represent the mean ± SD, ^a^P<0.05 vs. 5.5 mM glucose,^ b^P<0.05 vs. 30 mM glucose.

**Figure 6:**
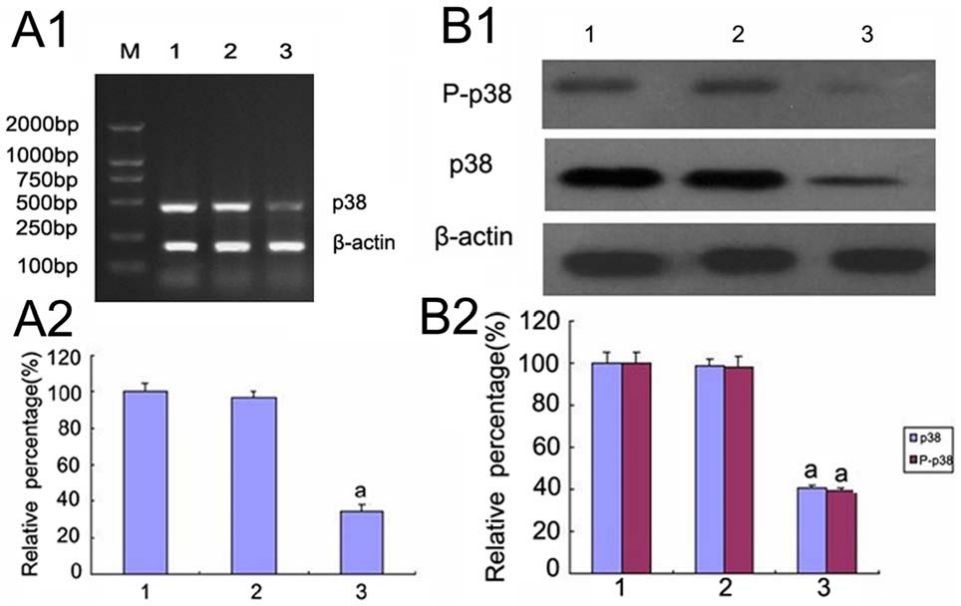
Effect of knockdown of p38MAPK by p38 MAPK siRNA on p38 MAPK expression by Western blot and RT-PCR analysis. (A)P38 MAPK expression were determined by RT-PCR of the HK-2 cells cultured for 72 h in 5.5 mM glucose (lane 1), 5.5 mM glucose+Cont siRNA (lane 2), 5.5 mM glucose+p38 siRNA (lane 3). (B)P38 MAPK expression were determined by Western blot of the HK-2 cells cultured for 72 h in 5.5 mM glucose (lane 1), 5.5 mM glucose+Cont siRNA (lane 2), 5.5 mM glucose+p38 siRNA (lane 3).Values represent the mean ± SD, ^a^P<0.05 vs. 5.5 mM glucose.

**Figure 7:**
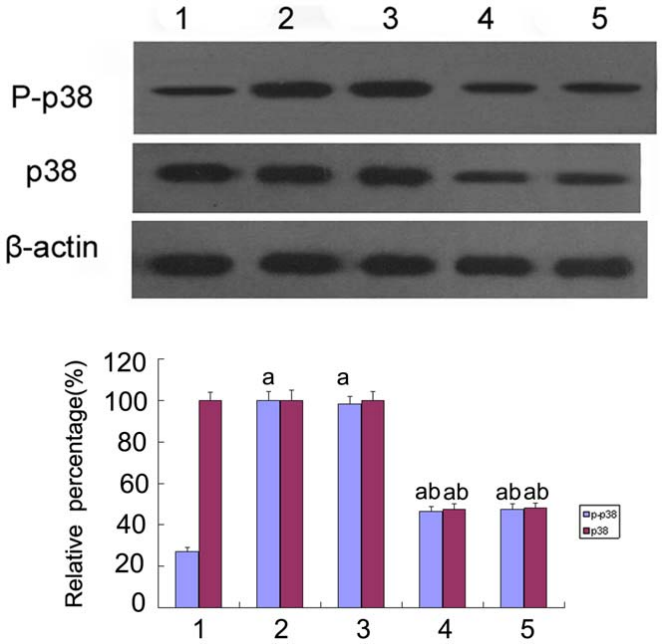
The expression of p38 MAPK and phosphorylated P38 MAPK by Western blot analysis. p38 MAPK and phosphorylated p38 MAPK were measured by Western blot of the HK-2 cells cultured in 5.5 mM glucose (lane 1), 30 mM glucose (lane 2), 30 mM glucose+Cont siRNA(lane 3), 30 mM glucose+p38 siRNA for 24 h(lane 4), 30 mM glucose+p38 siRNA for 48 h(lane 5). Values represent the mean ± SD, ^a^P<0.05 vs. 5.5 mM glucose, ^b^P<0.05 vs. 30 mM glucose.

To confirm whether high glucose could induce the epithelial-mesenchymal transition in the HK-2 cells, the cells were treated with low glucose (5.5 mM glucose) plus TGF-β1 or high glucose (30 mM glucose). We found that an increase in TGF-β1 of the HK-2 cells was induced by high glucose (Figure 1). The cells that were cultured in 5.5 mM glucose showed a typical epithelial cuboidal shape with the characteristic cobblestone morphology and abundant microvilli and mitochondria; however, the cells that were exposed to high glucose displayed distinct morphological changes (Figure 2 and 3). The expression of the epithelial phenotypic markers, E-cadherin and cytokeratin, as well as the mesenchymal phenotypic markers, α-SMA and vimentin, were determined (Figures 4 and 5). Under low glucose conditions, p38 MAPK gene silencing significantly inhibited the expression and phosphorylation of p38 MAPK (P<0.05) (Figure 6). To investigate the role of p38 MAPK in the high glucose-induced EMT, p38 MAPK and phosphorylated p38 were determined. The expressions of p38 MAPK under high glucose conditions were of no significant difference compared with that cultured in low glucose. But high glucose increased the phosphorylation of p38 MAPK in HK-2 cells. Knockdown of p38 gene lead to decrease of both expression and phosphorylation of p38 MAPK, the latter of which was induced by high glucose (P<0.05) (Figure 7).

### The role of p38 MAPK in the expression of E-cadherin, cytokeratin, vimentin, and α-SMA under high glucose conditions

**Figure 8:**
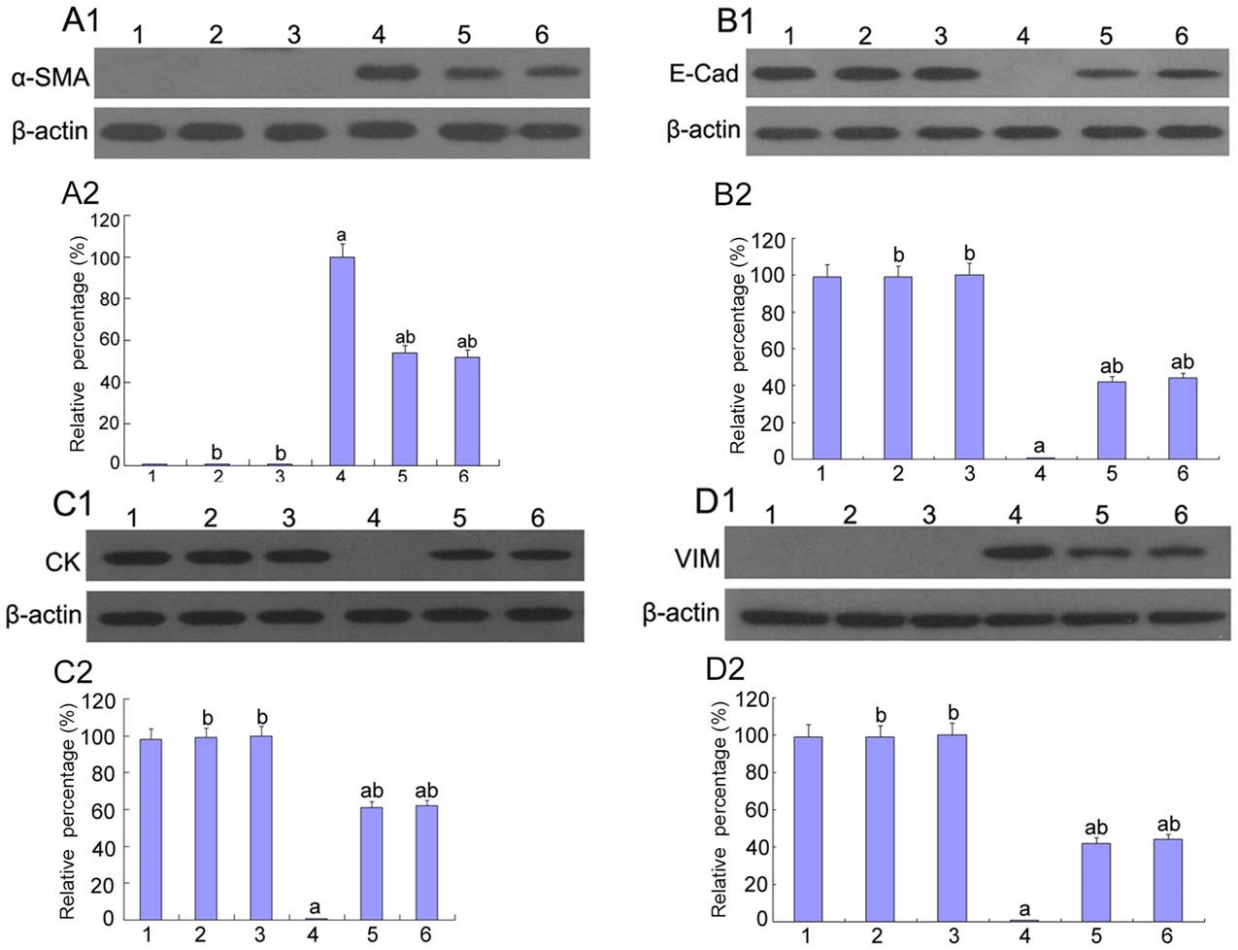
Expression of E-cadherin, CK, α-SMA, vimentin in HK-2 cells by Western blot. (A)α-SMA, (B)E-caderin, (C)CK, (D)vimentin were measured by Western blot of the HK-2 cells cultured in 5.5 mM glucose (lane 1), 5.5 mM glucose with p38 siRNA (lane 2), 5.5 mM glucose with AP-1 inhibitor (lane 3), 5.5 mM glucose+TGF-β1 (10 ng/ml) (lane 4), 5.5 mM glucose+TGF-β1(10 ng/ml) with p38 siRNA (lane 5), 5.5 mM glucose+TGF-β1(10 ng/ml) with the AP-1 inhibitor (lane 6). ^a^P<0.05 vs. 5.5 mM glucose, ^b^P<0.05 vs. 5.5 mM glucose+TGF-β1.

E-cadherin is a Ca^2+^-dependent cell adhesion molecule that plays a significant role in the maintenance of renal epithelial polarity. Cytokeratins are proteins of keratin-containing intermediate filaments that are found in the intracytoplasmic cytoskeleton of the cells of epithelial tissue. Loss of E-cadherin and CK expression is considered to be the early changes in TGF-β-induced EMT [32]. High glucose or low glucose with TGF-β1 decreased the expression of E-cadherin and CK, but the expression of those proteins increased after pre-treatment with p38 siRNA (Figures 4, 5, 8). In addition, CK mRNA was down-regulated under high glucose conditions but was significantly up-regulated after pre-treatment with p38 siRNA (P<0.05) (Figure 5).

α-SMA and vimentin are cytoskeletal proteins found in fibroblasts but not in TECs. In cells cultured with 5.5 mM glucose, α-SMA and vimentin were not detected. However, high glucose or low glucose plus TGF-β1 resulted in a significant increase in α-SMA and vimentin expression, which was blocked by pre-treatment with p38 siRNA (P<0.05) (Figures 4, 5, 8). It was further confirmed that the vimentin mRNA levels were significantly increased when the cells were treated with high glucose, but these levels were reduced after pre-treatment with p38 siRNA (P<0.05) (Figure 5).

### Effect of p38 MAPK on fibronectin synthesis in HK-2 cells

**Figure 9:**
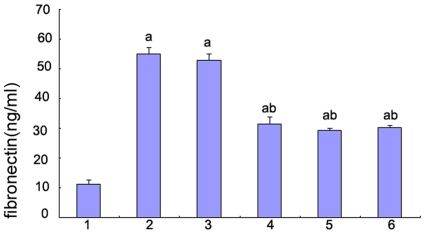
Fibronectin synthesis in HK-2 cells by ELISA. Fibronectin was measured by ELISA of the HK-2 cells in 5.5 mM glucose (lane 1), 30 mM glucose(lane 2), 30 mM glucose+Cont siRNA(lane 3), 30 mM glucose+p38 siRNA for 24 h(lane 4), 30 mM glucose+p38 siRNA for 48 h(lane 5) and 30 mM glucose+AP-1 inhibitor(lane 6). Values represent the mean ± SD, ^a^P<0.05 vs. 5.5 mM glucose, ^b^P<0.05 vs. 30 mM glucose.

Fibronectin is a high-molecular-weight extracellular matrix glycoprotein that binds to membrane-spanning receptor proteins and therefore plays a major role in cell adhesion, growth, migration and differentiation. Fibronectin is also central to the processes of wound healing and embryonic development. We detected fibronectin synthesis in HK-2 cells that were cultured in high glucose for 48 hours. Secretion of fibronectin was significantly up-regulated by the stimulation of high glucose (P<0.05), whereas the level of secretions was markedly decreased after pre-treatment with p38 siRNA (P<0.05) (Figure 9).

### The activation of AP-1 in high-glucose-treated HK-2 cells

**Figure 10:**
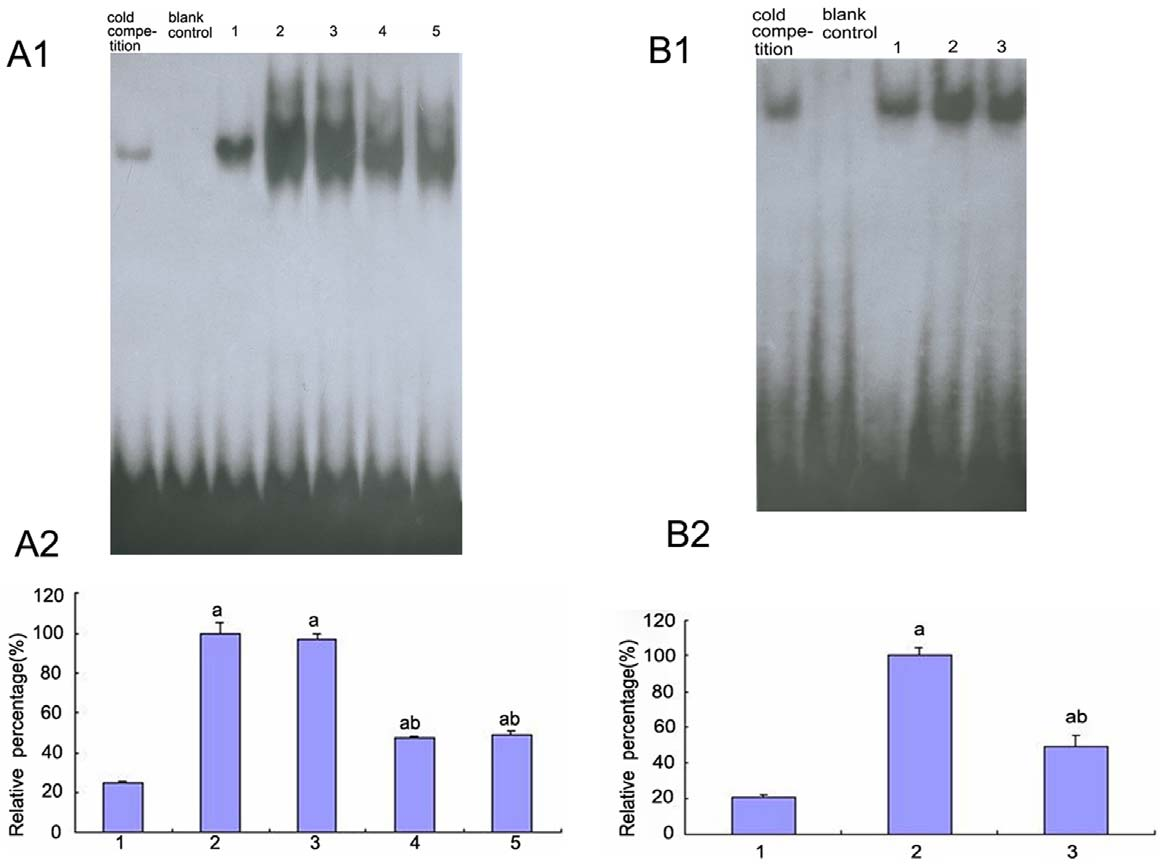
Activity of AP-1 in HK-2 cells by EMSA. (A)AP-1 activity was analyzed by EMSA of the HK-2 cells cultured in 5.5 mM glucose (lane 1), 30 mM glucose(lane 2), 30 mM glucose+Cont siRNA(lane 3), 30 mM glucose+p38 siRNA for 24 h (lane 4), 30 mM glucose+p38 siRNA for 48 h(lane 5).(B) AP-1 activity was analyzed by EMSA of the HK-2 cells cultured in 5.5 mM glucose(lane 1), 30 mM glucose (lane 2) and 30 mM glucose+AP-1 inhibitor(lane 3). Values represent the mean ± SD, ^a^P<0.05 vs. 5.5 mM glucose, ^b^P<0.05 vs. 30 mM glucose.

**Figure 11:**
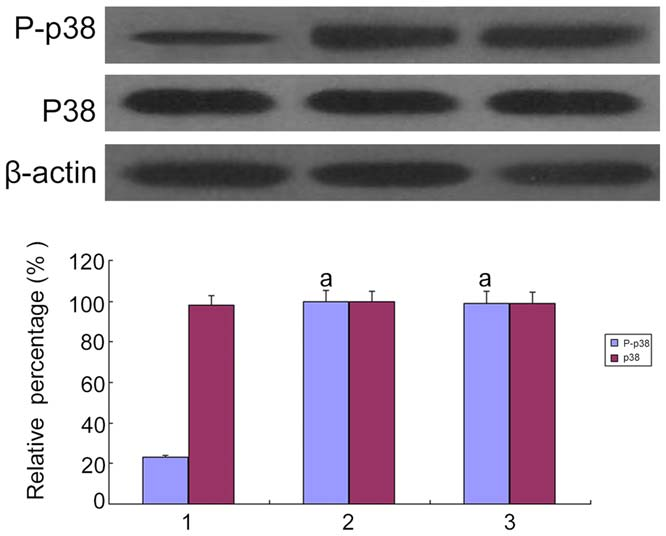
Expression and phosphorylation of p38 MAPK in HK-2 cells by Western blot. Expression and phosphorylation of p38 MAPK under 5.5 mM glucose (lane 1), 30 mM glucose(lane 2) and 30 mM glucose+AP-1 inhibitor were determined by. ^a^P<0.05 vs. 5.5 mM glucose.

Because MAPKs have been shown to play a critical role in the up-regulation of AP-1 activity in various types of cultured cells via multiple mechanisms, we attempted to examine the possible involvement of p38 MAPK, which is the major upstream kinase, in AP-1 activation in HK-2 cells. The levels of AP-1 binding activity increased after treatment with high glucose but decreased after transfection with p38 siRNA (P<0.05) (Figure 10). To further study the transactivation of AP-1 in high-glucose-treated HK-2 cells, experiments using [6]-Gingerol [33], which is a chemical inhibitor of AP-1, demonstrated that it could partially inhibit high-glucose-induced activation of AP-1, but was irrelevant to the regulation of p38 expression and phosphorylation. (P<0.05) (Figure 10 and 11).

### The effect of AP-1 on E-cadherin, cytokeratin, vimentin, and α-SMA expression under high glucose conditions

**Figure 12:**
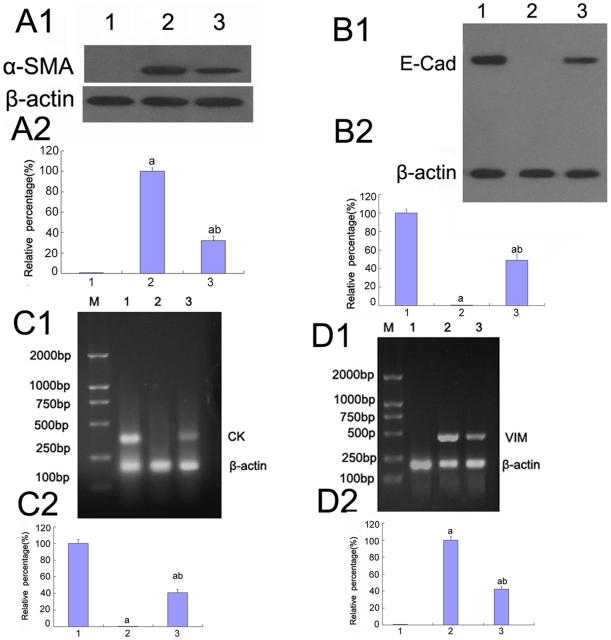
Expression of E-cadherin, CK, α-SMA, vimentin in HK-2 cells by Western blot and RT-PCR analysis. (A–B)α-SMA(A) and E-cadherin(B) were detected by Western blot of the HK-2 cells incubated in 5.5 mM glucose (lane 1), 30 mM glucose(lane 2), 30 mM glucose+AP-1 inhibitor(lane 3).(C–D)mRNA of CK(C) and vimentin(D) was measured by RT-PCR of the HK-2 cells incubated in 5.5 mM glucose (lane 1), 30 mM glucose(lane 2), 30 mM glucose+AP-1 inhibitor(lane 3). Values represent the mean ± SD. ^a^P<0.05 vs. 5.5 mM glucose, ^b^P<0.05 vs. 30 mM glucose.

To determine the role of AP-1 in the EMT, E-cadherin, CK, vimentin, and α-SMA expression was assessed. Compared to cells that were incubated with 5.5 mM glucose, the expression of E-cadherin and CK was significantly decreased under high glucose conditions or low glucose with TGF-β1 but was increased after pre-treatment with the AP-1 inhibitor, protein expression was increased (P<0.05) (Figure 4, 8, 12). It was further confirmed that the CK mRNA levels were markedly diminished under high glucose conditions; however, the levels were notably increased by pre-treatment with the AP-1 inhibitor (P<0.05) (Figure 12).

α-SMA and vimentin were not detected in cells that were incubated with 5.5 mM glucose. After culture with high glucose or low glucose with TGF-β1, the expression of α-SMA and vimentin were markedly increased but decreased with pre-treatment with the AP-1 inhibitor (P<0.05) (Figure 4, 8, 12). The levels of vimentin mRNA were increased when the cells were treated with high glucose, whereas they were significantly decreased after pre-treatment with the AP-1 inhibitor (P<0.05) (Figure 12).

### The effect of AP-1 on fibronectin synthesis in HK-2 cells

In the culture media from the cells treated with high glucose, the concentration of fibronectin was significantly greater than that of the media from the cells treated with 5.5 mM glucose; the concentration of fibronectin after pre-treatment with the AP-1 inhibitor was significantly decreased compared to the concentration after treatment with high glucose (Figure 9).

### The role of p38 MAPK/AP-1 in Snail expression under high glucose conditions

**Figure 13:**
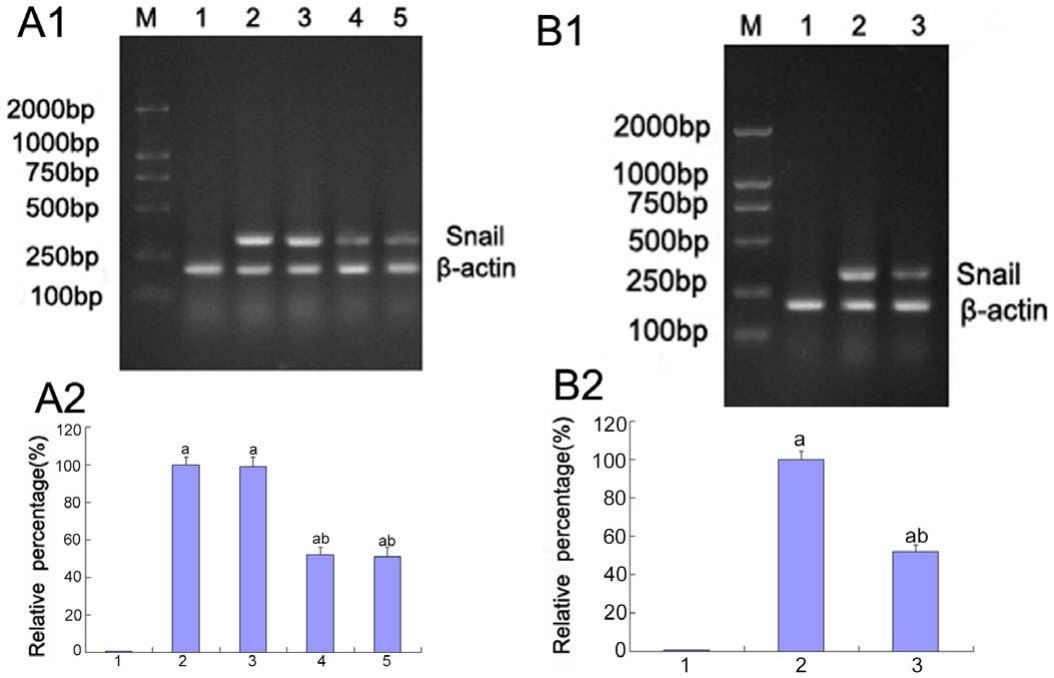
The expression of Snail mRNA in HK-2 cells by RT-PCR. (A)The levels of Snail mRNA were detected by RT-PCR of the HK-2 cells cultured in 5.5 mM glucose (lane 1), 30 mM glucose (lane 2), 30 mM glucose+Cont siRNA (lane 3), 30 mM glucose+p38 siRNA for 24 h (lane 4), 30 mM glucose DMEM+p38 siRNA for 48 h(lane 5).(B)The levels of the Snail mRNA were detected by RT-PCR of the HK-2 cells incubated in 5.5 mM glucose (lane 1), 30 mM glucose (lane 2), and 30 mM glucose+AP-1 inhibitor (lane 3). Values represent the mean ± SD, aP<0.05 vs. 5.5 mM glucose, bP<0.05 vs. 30 mM glucose.

Snail is a member of the Snail family of zinc finger transcription factors that induce the EMT initially identified as a gene controlling cell migration in the neural crest and developing mesoderm in the chick embryo [34]. Snail mRNA represses CDH1/E-cadherin and other epithelial genes, and induces the expression of mesenchymal genes in epithelial cells of diverse origin [35]–[37]. Therefore, we observed the effect of p38 MAPK and AP-1 on Snail mRNA expression in high glucose-induced EMT. In our study, Snail mRNA expression was amplified by the stimulation of high glucose compared to 5.5 mM glucose (P<0.05), whereas Snail mRNA expression was significantly decreased after pre-treatment with p38 siRNA or the AP-1 inhibitor (P<0.05) (Figure 13).

## Discussion

TIF has been recognized as a common terminal pathway for almost all forms of chronic kidney disease. TECs are pivotal in the processes of TIF through EMT because TECs are considered to be an essential source of myofibroblasts, which promote ECM synthesis [38], [39]. Increasing evidence indicates that TECs, which are the major constituents of the renal parenchyma, are not innocent bystanders but play a decisive role in the evolution of renal fibrosis. Recent clinical studies have also demonstrated the existence of EMT in human renal biopsies [40].

p38 MAPK activation is a key modulator in the progression of renal diseases and is thought to occur in various kinds of cells, including mesangial cells [41] and vascular endothelial cells [42]; however, little is known about the activation of p38 MAPK in renal tubular epithelial cells. Currently, there is a wealth of data that supports the central role of the p38 MAPK signaling pathway in high glucose-induced cell damage [43]–[46]. Therefore, We focused on the p38 MAPK signaling pathway to determine whether it mediates high glucose-induced EMT. Becasuse the activation of TGF-β signaling is sufficient to induce EMT in cultured epithelial cells and MAPK signaling pathways have been implicated inTGF-β-induced EMT [47], expression of TGF-β1 under high glucose conditions were detected to further confirm whether EMT could be induced by high glucose. The results showed that high glucose could induce EMT in human renal epithelial cells, which became more elongated and less adhered and lost their apical-to-basal polarity. During this process, cells lost E-cadherin and CK, showed increased de novo expression of α-SMA and vimentin and synthesized extracellular matrix molecules, such as fibronectin, which are features that are consistent with a more fibroblast-like phentoype. The activation of myofibroblasts plays a critical role in the processes of cell adhesion, actin re-organization and enhanced cellular progression of chronic renal disease. The role of p38 MAPK in tubular EMT is further supported by the p38 siRNA findings. In this study, p38 siRNA was shown to reverse the loss in E-cadherin and CK, suppress the expression of α-SMA and vimentin and inhibit fibronectin synthesis: these effects limited the development of the high glucose-induced epithelial mesenchymal transition. More intrestingly, increased apoptosis were also observed in cells in high glucose conditions for 48 h (Figure S1). The underlying mechanism of this high glucose-induced apoptosis is to be further elucidated.

Because MAPKs have been shown to play an important role in the up-regulation of AP-1 activation in various types of cultured cells via multiple mechanisms, we attempted to examine the possible involvement of p38 MAPK, which is the major upstream kinase, in the activation of AP-1 in HK-2 cells. AP-1 activity is rapidly induced by a broad range of stimuli, including growth factors, cytokines, stress signals, and infections, as well as oncogenic stimuli; therefore, AP-1 mediates many physiological processes, such as proliferation, survival, differentiation, apoptosis, and transformation [48]. The role of the AP-1 transcriptional complex in EMT has received little attention in the past and to our knowledge, data on the function of AP-1 in high glucose-induced EMT are lacking. The results of the present study provide strong evidence to support the role of AP-1 in high glucose-induced EMT. AP-1 has been shown to be a potential downstream therapeutic target in p38 MAPK-mediated diseases [49]. The findings in our study included the induction of AP-1 under high glucose conditions and its partial abrogation mediated by p38 siRNA. The levels of AP-1 binding activity were increased under high glucose conditions but were decreased after transfection with p38 siRNA. To further study AP-1 transactivation in high glucose-induced EMT, experiments demonstrated that [6]-Gingerol, which is a chemical inhibitor of AP-1, inhibited high glucose-induced activation of AP-1. With high glucose treatment, HK-2 cells underwent striking morphological alterations, including membrane ruffling, numerous pseudopodial protrusions, increased cell motility and anchorage-independent (invasive) growth. These changes were inhibited by pre-treatment with [6]-Gingerol.

The Snail family members have been shown to be involved in the regulation of EMT during embryonic development and tumor progression [50]. Snail possesses DNA binding capacity and recognizes the E-box elements in the promoter region of its target genes, which include E-cadherin. Binding of Snail to its cognate E-box often leads to the suppression of gene transcription [51], [52]. Earlier studies showed that induction of Snail, which commonly occurs in malignant carcinomas, suppresses epithelial E-cadherin expression [53], [54]. In this study, Snail expression was promoted in HK-2 cells under high glucose conditions, but knockdown of p38 MAPK and pretreatment with [6]-Gingerol reversed the increased Snail expression, which suggests that Snail expression in vitro is dependent on the p38/AP-1 signaling pathway. The expression of Snail suppressed the expression of E-cadherin protein. Although the underlying mechanism of Snail inhibition remains to be elucidated, the finding that p38/AP-1 is able to target Snail, which is a key EMT-regulatory gene, highlights a fundamental step in the p38/AP-1 signaling pathway in high glucose-induced EMT, which may prove its role in TIF.

This study provides a novel insight into the role of the p38/AP-1 signaling pathway, which was shown to be a crucial step in high glucose-induced EMT in HK-2 cells. The results indicate that the striking changes in TECs were induced by high glucose; these changes include the following: the activation of phosphorylated p38 MAPK and AP-1; alterations in cell morphology; loss of the epithelial markers, E-cadherin and CK; de novo expression of vimentin and α-SMA; and an increase in fibronectin levels. The siRNA-mediated down-regulation of p38 expression prevented AP-1 activation and the high glucose-induced EMT. In addition, the suppression of the AP-1 transcriptional complex blocked the high glucose-induced EMT. The inhibition of the p38/AP-1 signaling pathway limited the development of the EMT in vitro and suppressed the expression of Snail, which is a key EMT-regulatory gene. Taken together, this study suggests the p38 MAPK pathway may play an important role in high glucose-induced EMT through the activation of AP-1 in tubular epithelial cells.

## Materials and Methods

### Antibodies and chemical inhibitors

Anti-phospho-p38 antibodies and anti-p38 antibodies were purchased from Cell Signaling Technology (USA). Anti-E-cadherin, anti-α-SMA, anti-vimentin, anti-CK and anti-TGF-β1 antibodies were purchased from Santa Cruz Biotechnology, Inc. (USA), and [6]-Gingerol was purchased from Wako Ltd. (Japan).

### Cell culture and experimental conditions

Experiments were performed with HK-2 cells (American Type Culture Collection number CRL-2190), which are human proximal tubular epithelial cells immortalized by transduction with human papillomavirus type 16 E6/E7 genes. Cells were cultured in Dulbecco's modified Eagle's medium (low glucose) (Gibco, UK) that was supplemented with 10% fetal calf serum (Sigma, USA). The cells were seeded at 1.5×10^6^ cells/10 cm diameter dishes for 3–4 days at 37°C in a humidified atmosphere containing 5% CO_2_.

### Transfection of siRNA

To silence the p38 MAPK gene expression, siRNA oligonucleotides with the following sequences were designed: p38 siRNA, 5′-GAAGCTCTCCAGACCATTT-3′ and negative Cont-siRNA, 5′ -GACTTCATAAGGCGCATGC-3′. The HK-2 cells were cultured for 24 h before the transfection. After a 24-h or 48-h incubation with p38 siRNA or a 24-h incubation with Cont-siRNA, the cells were cultured with high glucose (30 mM glucose) for an additional 48 h. Cells that were not transfected and were exposed to normal glucose (5.5 mM glucose) or high glucose (30 mM glucose) for 48 h were considered to be the controls. Transfection was processed in 24-well plates with 40 nM siRNAs using Lipofectamine2000 (Invitrogen). p38 MAPK knockdown was confirmed by Western blot analysis and reverse transcription-PCR (RT-PCR).

### SDS-PAGE and Western blot analysis

Cells were washed twice with PBS and lysed with 2X SDS-PAGE sample buffer. Next, 50 µg of total protein was separated by 10% SDS-PAGE and transferred to nitrocellulose membranes; the membranes were then blocked with 1% polyvinylalcohol in PBS containing 0.2% Tween 20 for 10 min and incubated at 4°C overnight with primary antibodies (diluted to various concentrations in blocking buffer (5% or 1% skim milk in PBS-Tween (0.2% Tween 20)) against the following target proteins: p38, phospho-p38 (1∶1000), E-cadherin (1∶200), α-SMA (1∶200), CK (1∶200), Vimentin (1∶600), TGF-β1 (1∶500) and β-actin (1∶2000). The membranes were then washed three times with PBS-Tween for 10 min and incubated with species-specific peroxidase-conjugated secondary antibodies (Santa Cruz, CA) diluted in blocking buffer (5% skim milk in PBS-Tween) for 2 h at room temperature. Specific bands were detected using the ECL system (Amersham) and the Bio-Rad electrophoresis image analyzer (Bio-Rad, Hemel Hampstead, UK).

### RNA extraction and reverse transcription

Total RNA was extracted from the HK-2 cells using the Tripure reagent (Roche, Germany) according to the manufacturer's instructions. RNA samples were qualified by the measurement of the optic absorbance at 260 and 280 nm with a resultant A260/A280 ratio that ranged from 1.8 to 2.0, which indicated a high purity of the extracted RNA. The concentration of total RNA was calculated according to A260. Aliquots of total RNA (1.0 µg each) from each sample were reverse transcribed into cDNA according to the instructions of the First Strand cDNA Synthesis Kit (Takara, China).

### Reverse Transcriptase-PCR

Semiquantitative reverse transcriptase-PCR (RT-PCR) was used to determine the steady-state mRNA levels of Snail, CK and vimentin. Briefly, after reverse transcription of renal total RNA, cDNA was used as a template for the PCR reactions using gene-specific primer pairs. Generally, 30 to 35 cycles for amplification in the linear range were used. Quantification of PCR products was performed using densitometry, and the relative mRNA levels were calculated and compared after normalization to beta-actin. The primers for the RT-PCR reaction were purchased from Sangon Biotech (China). The sequences were as follows: Snail (human), sense 5′-CATTC CACGCCCAGCTACCC-3′ and antisense 5′-CGCCCAGGCTC ACATATTCC-3′; CK mRNA, sense 5′-CCCAGAGCCTTGAGATAGAA C-3′ and antisense 5′-CACGACCTTGCCATCCAC-3′; vimentin mRNA, sense 5′-CGCTTCGCCAACTACAT C-3′ and antisense 5′-GGTCAGGCTT GGAAACATC-3′; and beta-actin, sense 5′-CACCAACTGGGACG ACAT-3′ and antisense 5′-AC AGCCTGGATAGCAACG-3′.

### Transmission electron microscopy

For transmission electron microscope analysis, HK-2 cells were harvested, pre-fixed with 2% glutaraldehyde for 2 h at 4°C, washed twice with PBS, and then post-fixed with 1% osmic acid for 2 hours at 4°C. After an additional two washes in PBS, the samples were dehydrated with an ethanol gradient, washed twice with propylene oxide, soaked in ethoxyline resin overnight, and mounted at 60°C for 48 hours. Thin sections were cut with an ultramicrotome and then viewed under a transmission electron microscope (Hitachi, Japan).

### ELISA (enzyme-linked immunosorbent assay)

Fibronectin release was determined using a Fibronectin ELISA kit (Jingmei BioTech, China). After exposure to the defined experimental conditions, culture supernatants were harvested. Different concentrations of supernatants were added to 96-well plates for 2 h at 37°C, followed by three washes with PBST buffer. Next, 100 µl of peroxidase-conjugated IgG (diluted with PBS to a concentration of 1∶5000) was added to each well, and the plates were incubated at 25°C for 2 h. After the plates were washed three times with PBST, 100 µl of substrate solution (3, 3′, 5, 5′-tetramethylbenzidine, TMB) was added to each well using a multichannel pipet. The reaction was stopped by adding 50 µl of 2.0 M sulfuric acid to each well. After sufficient color development, absorbance values were immediately determined with an ELISA reader at a wavelength of 450 nm.

### Immunocytochemistry

Cells under different conditions were plated onto different six-well plates at a density of 1×10^5^ cells/well; each well contained a polylysine-coated slide. Cells were fixed in 4% PFA for 15 min at 4°C and individually stained with monoclonal antibodies to E-cadherin, α-SMA, vimentin and CK using immunocytochemistry according to the instructions in the immunocytochemical detection kit (Zhongshan Goldbridge Biotech, China). Briefly, HK-2 cells were rinsed in phosphate-buffered saline, fixed in 2% paraformaldehyde, preincubated with 10% fetal calf serum to block nonspecific binding, and then incubated with antibodies for 60 min. After washing with phosphate-buffered saline, endogenous peroxidase was inactivated by incubation with 0.3% H_2_O_2_ in methanol for 15 min; this was followed by incubation with peroxidase-conjugated IgG and peroxidase-conjugated anti-peroxidase complexes and color development with diaminobenzidine to produce a brown color. All procedures were performed at room temperature. Data from five experiments were expressed as the mean percentage.

### EMSA

The nuclear extracts were prepared from the cells under the different conditions. The EMSA was performed using the LightShift chemiluminescent EMSA kit (Pierce, Rockford, USA) according to the manufacturer's instructions. Briefly, the biotin-labeled probes were incubated with the nuclear extracts for 30 min at 37°C, and the reaction samples were electrophoresed on a 4% non-denaturing acrylamide gel in a 0.5X Tris-borate-EDTA buffer. After transfer to nylon membranes, the biotin-labeled probes were detected. The following AP-1 probe was used for the EMSA: AP-1 response element (AP1, 5′CGC TTG ATG AGT CAG CCG GAA3′)

### Data analysis

All experiments were repeated at least three times. Values were reported as mean ± SD. Data were analyzed using SPSS 13.0 software. Statistical significance was assessed using ANOVA and LSD-t test, and P values of less than 0.05 were considered to be statistically significant.

## Supporting Information

Figure S1 HK-2 cells apoptosis were measured by flow cytometry. (A) shows the rate of apoptosis in HK-2 cells under 5.5 mM glucose conditions for 48 h (lane 1), (B) shows the rate of apoptosis in HK-2 cells under 30 mM glucose conditions for 48 h (lane2).
(TIF)

## References

[1] Zeisberg M, Kalluri R (2004). The role of epithelial-to-mesenchymal transition in renal fibrosis.. J Mol Med.

[2] Masayuki I, David P, Theodore MD, Chengsen X, Hirokazu O (2002). Evidence that fibroblasts derive from epithelium during tissue fibrosis.. J Clin Invest.

[3] Zeisberg M, Strutz F, Muller GA (2000). Role of fibroblast activation in inducing interstitial fibrosis.. J Nephrol.

[4] Yang J, Liu Y (2001). Dissection of key events in tubular epithelial to myofibroblast transition and is implications in renal interstitial fibrosis.. Am J Pathol.

[5] Massagúe J (1998). TGF-β Signal Tranduction.. Annu Rev Biochem.

[6] Paivi JM, Reinhard E, Alfredo RL, Rik D (1994). TGF-β Induced Transdifferentiation of Mammary Epithelial Cells to Mesenchymal Cells: Involvement of Type I Receptors.. J Cell Biol.

[7] Ester P, Aristidis M, Akira K, Carl-Henrik H, Peter TD (1999). TGF-β type I receptor/ALK-5 and Smad proteins mediate epithelial to mesenchymal transdifferentiation in NMuMG breast epithelial cells J Cell Sci.

[8] Erwin PB, Markus B (2002). TGF-β Signaling in Renal Disease.. J Am Soc Nephrol.

[9] Koh O, Jiahuai H (2000). The p38 signal transduction pathway Activation and function.. Cell Signal.

[10] John MK, Joseph A (1996). Sounding the alarm: protein kinase cascades activated by stress and inflammation.. J Biol Chem.

[11] Cano E, Mahadevan LC (1995). Parallel signal processing among mammalian MAPKs.. Trends Biochem Sci.

[12] Tomlinson DR (2003). Mitogen activated protein kinase as glucose transducers for diabetic complications.. Diabetologia.

[13] Tanaka T, Kanai H, Sekiguchi K, Aihara Y, Yokoyama T (2000). Induction of VEGF gene transcription by IL-1 beta is mediated throughstress-activated MAP kinases and Sp1 sites in cardiac myocytes.. J Mol Cell Cardiol.

[14] Jung YD, Liu W, Reinmuth N, Ahmad SA, Fan F (2001). Vascular endothelial growth factor is upregulated by interleukin-1 beta in human vascular smooth muscle cells via the p38 mitogen-activated protein kinase pathway.. Angiogenesis.

[15] Duyndam MC, Hulscher ST, van der Wall E, Pinedo HM, Boven E (2003). Evidence for a role of p38 kinase in hypoxia-inducible factor-1-independent induction of vascular endothelial growth factor expression by sodium arsenite.. J Biol Chem.

[16] Tokuda H, Hatakeyama D, Shibata T, Akamatsu S, Oiso Y (2003). p38 MAP kinase regulates BMP-4-stimulated VEGF synthesis via p70 S6 kinase in osteoblasts.. Am J Physiol Endocrinol Metab.

[17] Tsai PW, Shiah SG, Lin MT, Wu CW, Kuo ML (2003). Up-regulation of vascular endothelial growth factor C in breast cancer cells by heregulin-beta1. A critical role of p38/nuclear factor-kappa B signaling pathway.. J Biol Chem.

[18] Wilmer WA, Dixon CL, Hebert C (2001). Chronic exposure of human mesangial cells to high glucose environments activates the p38 MAPK pathway.. Kidney Int.

[19] Gruden G, Zonca S, Hayward A, Thomas S, Maestrini S (2000). Mechanical stretch-induced fibronectin and transforming growth factor-beta1 production in human mesangial cells is p38 mitogen-activated protein kinase-dependent.. Diabetes.

[20] Kang SW, Adler SG, Nast CC, LaPage J, Gu JL (2001). 12-lipoxygenase is increased in glucose-stimulated mesangial cells and in experimental diabetic nephropathy.. Kidney Int.

[21] Hisayo F, Sayu O, Kenji I, Mariko H, Midori A (2004). ERK and p38 mediate high-glucose-induced hypertrophy and TGF-beta expression in renal tubular cells.. Am J Physiol Renal Physiol.

[22] Kathleen MM (2000). Role of Ras and Mapks in TGFβ signaling.. Cytokine Growth F R.

[23] Neil AB, Roy Z, Mayshan G, Maureen M, Harold LM (2001). Integrin β1 Signaling Is Necessary for Transforming Growth Factor-β Activation of p38MAPK and Epithelial Plasticity.. J Biol Chem.

[24] Hughes P, Dragunow M (1995). Induction of immediate-early genes and the control of neurotransmitter-regulated gene expression within the nervous system.. Pharmacol Rev.

[25] Morgan JI, Curran T (1991). Stimulus-transcription coupling in the nervous system: involvement of the inducible protooncogenes fos and jun.. Annu Rev Neurosci.

[26] Fatima M, Damien G, Moshe Y (2001). The mammalian Jun proteins: redundancy and specificity.. Oncogene.

[27] Wolfram J, Emmanuelle P, Erwin FW (2001). AP-1 in mouse development and tumorigenesis.. Oncogene.

[28] Shaulian E, Karin M (2002). AP-1 as a regulator of cell life and death.. Nat Cell Biol.

[29] Barría M, Droguett MA, Burgos ME, Ardiles LG, Flores C (2001). Tubular NF-kappaB and AP-1 activation in human proteinuric renal disease.. Kidney Int.

[30] Jung-Hee J, Young-Joon S (2005). AP-1 mediates beta-amyloid-induced iNOS expression in PC12 cells via the ERK2 and p38 MAPKsignaling pathways.. Biochem Bioph Res Co.

[31] Guo YS, Hellmich MR, Wen XD, Townsend CM (2001). Activator protein-1 transcription factor mediates bombesin-stimulated cyclooxygenase-2 expression in intestinal epithelial cells.. J Biol Chem.

[32] Zheng G, Lyons JG, Tan TK, Wang Y, Hsu TT (2009). Disruption of E-cadherin by matrix metalloproteinase directly mediates epithelial-mesenchymal transition downstream of transforming growth factor-beta1 in renal tubular epithelial cells.. Am J Pathol.

[33] Bode AM, Ma WY, Surh YJ, Dong Z (2001). Inhibition of epidermal growth factor- induced cell transformation and activator protein 1 activation by [6]-Gingerol.. Cancer Res.

[34] Nieto MA, Sargent MG, Wilkinson DG, Cooke J (1994). Control of cell behavior during vertebrate development by Slug, a zinc finger gene.. Sci.

[35] Héctor P, David O, Amparo C (2007). Snail, Zeb and bHLH factors in tumour progression: an alliance against the epithelial phenotype?. Nat Rev Cancer.

[36] Alves CC, Carneiro F, Hoefler H, Becker KF (2009). Role of the epithelial-mesenchymal transition regulator Slug in primary human cancers.. Front Biosci.

[37] César C, María PC, Carolina VD, Isidro SG (2007). Function of the zinc-finger transcription factor SNAI2 in cancer and development.. Annu Rev Genet.

[38] Strutz F, Muller GA (2000). Transdifferentiation comes of age.. Nephrol Dial Transpl.

[39] Kalluri R, Neilson EG (2003). Epithelial-mesenchymal transition and its implications for fibrosis.. J Clin Invest.

[40] Maria PR, Franco F, Laura G, Giacomo DA, Carlo G (2002). Epithelial-mesenchymal transition of tubular epithelial cells in human renal biopsies.. Kidney Int.

[41] Reddy MA, Adler SG, Kim YS, Lanting L, Rossi J (2002). Interaction of MAPK and 12-lipoxygenase pathway in growth and matrix protein expression in mesangial cells.. Am J Physiol Renal Physiol.

[42] Touyz RM, He G, El Mabrouk M, Diep Q, Mardigyan V (2001). Differential activation of extracellular signal-regulated protein kinase 1/2 and p38 mitogen activated-protein kinase by AT1 receptors in vascular smooth muscle cells from Wistar-Kyoto rats and spontaneously hypertensive rats.. J Hypertens.

[43] Tanaka T, Kanai H, Sekiguchi K, Aihara Y, Yokoyama T (2000). Induction of VEGF gene transcription by IL-1 beta is mediated through stress-activated MAP kinases and Sp1 sites in cardiac myocytes.. J Mol Cell Cardiol.

[44] Jung YD, Liu W, Reinmuth N, Ahmad SA, Fan F (2001). Vascular endothelial growth factor is upregulated by interleukin-1 beta in human vascular smooth muscle cells via the p38 mitogen-activated protein kinase pathway.. Angiogenesis.

[45] Tokuda H, Hatakeyama D, Shibata T, Akamatsu S, Oiso Y (2003). p38 MAP kinase regulates BMP-4-stimulated VEGF synthesis via p70 S6 kinase in osteoblasts.. Am J Physiol Endocrinol Metab.

[46] Tsai PW, Shiah SG, Lin MT, Wu CW, Kuo ML (2003). Up-regulation of vascular endothelial growth factor C in breast cancer cells by heregulin-beta1. A critical role of p38/nuclear factor-kappa B signaling pathway.. J Biol Chem.

[47] Jiri Z, Markus B, Dan L, Yaw-Ching Y, Aldo M (2001). Genetic programs of epithelial cell plasticity directed by transforming growth factor-b.. P Natl Acad Sci.

[48] Hess J, Angel P, Schorpp-Kistner M (2004). AP-1 subunits: quarrel and harmony among siblings.. J Cell Sci.

[49] Shiby P, Agnes MR, Hong JL, Yan J, Bandaru S (2009). Anti-inflammatory action of pterostilbene is mediated through the p38 MAPK pathway in colon cancer cells.. Cancer Prev Res.

[50] Nieto MA (2002). The snail superfamily of zinc-finger transcription factors.. Nat Rev Mol Cell Biol.

[51] Palmer HG, Larriba MJ, Garcia JM, Ordonez-Moran P, Pena C (2004). The transcription factor SNAIL represses vitamin D receptor expression and responsiveness in human colon cancer.. Nat Med.

[52] Pena C, Garcia JM, Silva J, Garcia V, Rodriguez R (2005). E-cadherin and vitamin D receptor regulation by SNAIL and ZEB1 in colon cancer:Clinicopathological correlations.. Hum Mol Genet.

[53] Batlle E, Sancho E, Franci C, Dominguez D, Monfar M (2000). The transcription factor snail is a repressor of E-cadherin gene expression in epithelial tumour cells.. Nat Cell Biol.

[54] Cano A, Perez-Moreno MA, Rodrigo I, Locascio A, Blanco MJ (2000). The transcription factor snail controls epithelial-mesenchymal transitions by repressing E-cadherin expression.. Nat Cell Biol.

