# Supplementary figures and images for: The Role of the p38 MAPK Signaling Pathway in High Glucose-Induced Epithelial-Mesenchymal Transition of Cultured Human Renal Tubular Epithelial Cells
Source: PLoS One. 2011 Jul 29;6(7):e22806. doi: 10.1371/journal.pone.0022806 (PMC3146517; doi:10.1371/journal.pone.0022806)

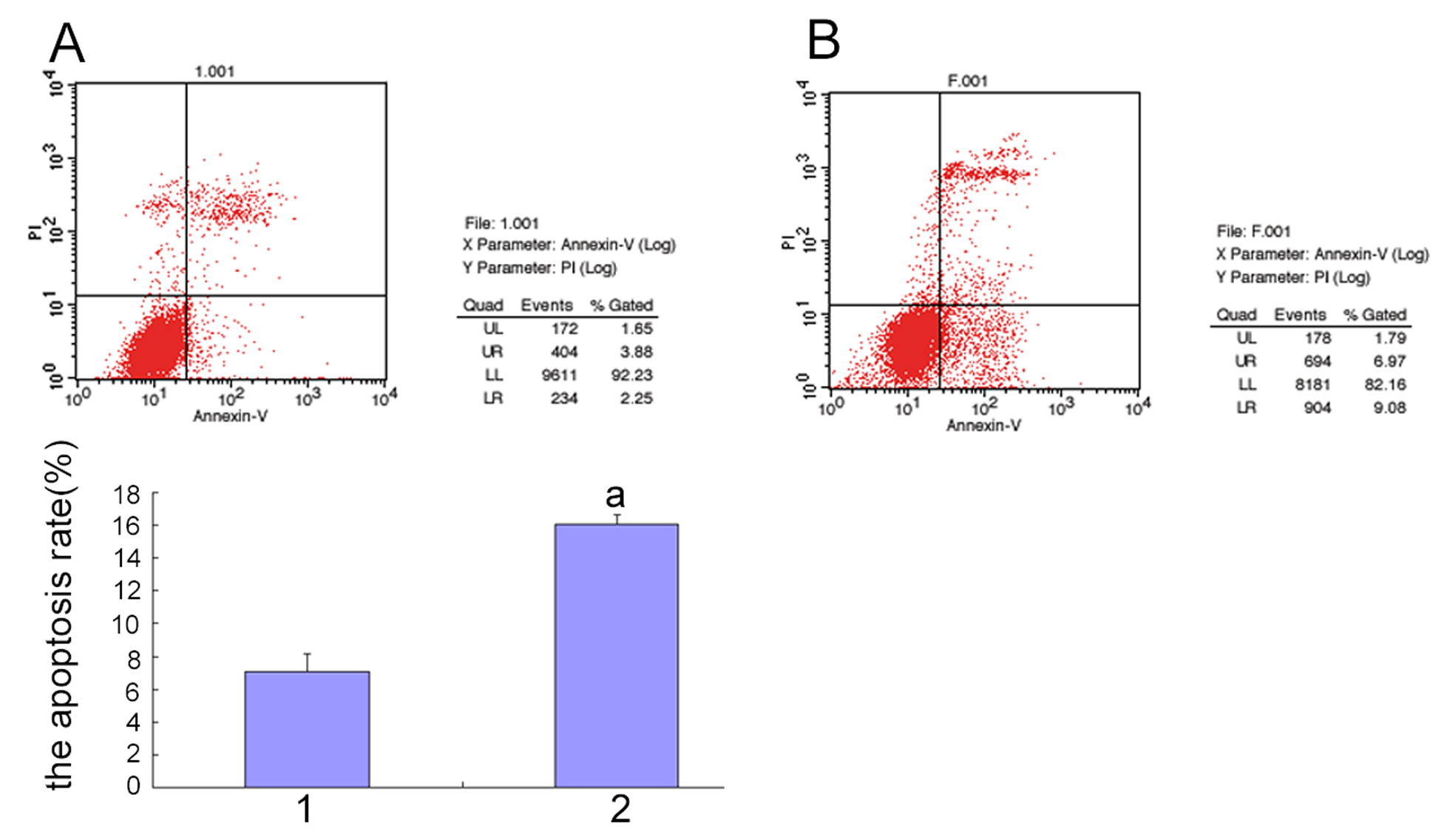

Supplement: Figure S1 — HK-2 cells apoptosis were measured by flow cytometry. (A) shows the rate of apoptosis in HK-2 cells under 5.5 mM glucose conditions for 48 h (lane 1), (B) shows the rate of apoptosis in HK-2 cells under 30 mM glucose conditions for 48 h (lane2). (TIF) [file pone.0022806.s001.tif]
